# The Plague in Suffolk

**Published:** 1910-11-05

**Authors:** 


### The Hospital

A JOURNAL OF

The Medical Sciences and Hospital Administration.

NEW SERIES. No. 195, VOL. VIII. [No. 1267, VOL. XLIX.] SATURDAY, NOVEMBER 5, 1910.

### THE PLAGUE IN SUFFOLK.

OUR immunity from really serious outbreaks of epidemic and highly fatal diseases is nowadays so nearly complete in Britain, that it comes as a startling and severe shock to learn that any of those catastrophic visitations which devastate parts of Asia now and then have got a footing on our coasts. In the case of plague, familiar to every school child from the accounts of the historical visitation of 1665-66, the awful mortality in our Indian Empire during the present century naturally accentuates the common uneasiness. It is decidedly a very serious matter that four deaths from this fell disease have occurred at Freston; and it is decidedly disquieting to be told that enormous numbers of rats, cats, rabbits, and other small game have died, and that the corpses swarm with plague bacilli. It is, again, of vast importance that effective measures shall be taken to prevent the spread of the infection, both among human beings and animals, and to inaugurate an active campaign against vermin of every kind, big and little. But it is also an occasion which should not give rise to anything approaching panic; the situation must be grappled with promptly and courageously, but with calmness and confidence as well.

It is to be recollected that there are fundamental differences between this country and India, which of themselves render it most unlikely—nay, impossible—that any outbreak of plague at all comparable to those which have there decimated the Bombay population should be apprehended here. In this country the brown Norway rat is the species which flourishes, not the smaller black rat which infests India. Luckily for us, the fleas which live upon the brown rat are a variety which does not attack man; so this, the most important, avenue of infection is closed as far as we are concerned. Moreover, the brown rat is a dweller in banks and ditches, in sewers, docks, and warehouses, not in dwelling-houses to any great degree. The black rat, on the contrary, loves the ill-constructed hovels of the native peasantry, and harbours parasites which are partial also to man. So that we start with a tremendous advantage from these facts alone. Then our system of sanitation, though admittedly not perfect, especially in country places, is yet comparatively complete, and affords few loopholes for the communication of the *bacillus pestis* from rat to man by direct transmission. Furthermore, the winter season now approaching is that in which both rats and fleas breed less freely than in warmer weather, though both these animals are free breeders.

By all means let rats be exterminated, whether infected or non-infected, whether in Suffolk or in any other part of the United Kingdom. The rat is an enemy whose inroads upon the national wealth must be, taken in the aggregate, enormous. For he exists in millions, and few kinds of food come amiss to him. That the rats are suffering severely from the plague and dying off in large numbers is an excellent thing of itself, so long as it results in a diminution of the rat population without any spread of the disease to mankind. And the organised rat hunts to which the population of the "Samford peninsula" is devoting itself are a form of practical hygiene whose utility is unquestionable. For most certainly there are too many rats in this country, and there is far too much slackness as a rule about rooting them out. Farmers suffer annual losses which would probably astonish them could they be accurately computed, yet are seldom energetic enough to deal as fully as they might with the rats. Therefore, by all means, success to the rat-exterminators and their enterprise.

The outbreak, however, is apparently not confined to rats. Cats and rabbits have been proved to have died of the same disease, and as the latter are extensively used as food in country districts, and exported also to the towns, the matter has a serious importance from a public health point of view. Several of the large landowners in the affected district in East Suffolk are setting a splendid example on their estates, and the Great Eastern Railway is helping too. The Lord Lieutenant of the county is taking the matter up warmly, and the inhabitants have been circularised by the local Urban District Councils to refrain for the present from eating hares, rabbits, and ground game. It is suggested by one local medical officer of health that the sale or handling of any of these animals should be prohibited, and some of the facts which have transpired tend to show the value of the suggestion. Thus a ferret has died of a disease which is now proved to be plague, after eating a rabbit; a cat, which was shot at Stutton because it was ill, has been disinterred, and it, too, is found to contain plague bacilli. So it is quite evident that the warnings issued against contact with these animals are not to be neglected with impunity. At the time of writing the infected area is still spreading, and threatens to involve a large part of East Suffolk; it is sincerely to be hoped that the public interest, which has been so rapidly aroused, will not be allowed to die down until the outbreak is thoroughly mastered, and that no further spread of the epidemic may have to be recorded. This consummation is one which should speedily be reached if once the co-operation of the rural population can be secured. The matter touches the latter so urgently that any reluctance to help will be a lasting disgrace.

Finally, we would draw the attention of those who may not have seen Mr. Stephen Paget's letter in the Press, to the pamphlet of the Research Defence Society on plague in India, which gives a full account not only of the actual disease, but of the best-informed views as to its prevention. For sevenpence Mr. Paget will send this pamphlet, post free; its author is Lieut.-Colonel Bannerman, M.D., D.Sc., Director of the Bombay Bacteriological Laboratory.

